# S_T_Mamba: A Novel Jinnan Calf Diarrhea Behavior Recognition Model Based on Sequence Tree Mamba

**DOI:** 10.3390/ani15182646

**Published:** 2025-09-09

**Authors:** Wangli Hao, Yakui Xue, Hao Shu, Bingxue Lv, Hanwei Li, Meng Han, Yanhong Liu, Fuzhong Li

**Affiliations:** 1 College of Software, Shanxi Agricultural University, Jinzhong 030801, China; xueyakui@sxau.edu.cn (Y.X.); 20233028@stu.sxau.edu.cn (H.S.); lifuzhong@sxau.edu.cn (F.L.); 2 College of Information Science and Engineering, Shanxi Agricultural University, Jinzhong 030801, China; 202430008@stu.sxau.edu.cn (B.L.); 202430801@stu.sxau.edu.cn (H.L.); 3 Hangzhou Economic Development Zone, Hangzhou Dianzi University, Hangzhou 310018, China; hanm@hdu.edu.cn

**Keywords:** minimum spanning tree, state space model, behavior recognition, sequence processing strategy

## Abstract

The efficient and precise recognition of diarrhea-related behaviors in Jinnan calves is crucial for ensuring their healthy development. Nevertheless, conventional behavior recognition techniques are often limited by a notable decline in performance when distinguishing between similar behavioral patterns. This paper proposes a novel behavior recognition model for Jinnan calf diarrhea, named S_T_Mamba (Sequence Tree Mamba). Specifically, S_T_Mamba incorporates a sequence processing strategy and a tree state space module (TreeSSM). The sequence processing strategy utilizes sequence as inputs to capture the temporal dependencies underlying the video. Additionally, the tree state space module is designed to extract and aggregate long-range pixel association features from video frames, enabling the effective recognition of subtle distinctions between similar behaviors. Therefore, the proposed model significantly enhances the performance of calf diarrhea behavior recognition. Experimental results indicate that the S_T_Mamba model achieves state-of-the-art performance in Jinnan calf diarrhea behavior recognition. Specifically, S_T_Mamba achieves 99.78% accuracy, outperforming existing popular models by 0.59% to 1.99%.

## Simple Summary

Calf diarrhea behavior is a reliable indicator of health status, and accurate behavior recognition is vital for effective health surveillance and management. However, current behavior recognition techniques often struggle to distinguish between similar behaviors, leading to a decrease in performance. This research proposes a new model, named S_T_Mamba, which leverages a sequence processing strategy and a tree state space module to capture temporal dependencies and long-range pixel associations in video frames. The model can effectively recognize subtle differences in calf diarrhea behavior. Consequently, the performance of calf diarrhea behavior recognition is enhanced by the S_T_Mamba model.

## 1. Introduction

In smart farming, recognizing the behavior of diarrhea in Jinnan calves is crucial. This protects the health of the calves and improves the efficiency of breeding management [1]. However, Jinnan calves frequently face health challenges, especially diarrhea. Therefore, it is essential to develop an effective recognition model to promptly address this prevalent issue.

Wearable sensors are valuable tools for monitoring animal behavior. They can recognize calf behaviors such as feeding, ruminating, drinking, locomotion, and resting [2]. However, accurate recognition remains a challenge. Dissanayake et al. [3] proposed a method using collar acceleration data. They used a personalized weighted AdaBoost (PWA) model. This model incorporates pairing, clustering, and adjusting training sample weights. Based on data from 21 calves, PWA showed better equilibrium accuracy than non-personalized models. Further, Zhang et al. introduced a digital twin system for dairy cows. They developed a lifecycle model that integrates indoor positioning and Inertial Measurement Unit (IMU) data. Five calves wore Ultra-Wideband IMU collars for data collection. Algorithms such as SVM, KNN, and LSTM were used for behavior recognition. Results showed that LSTM was most effective. In foraging behavior, Precision reached 91.05% with IMU data and 94.97% with location data [4,5].

Traditional methods rely on sensors and machine learning. However, these conventional approaches are increasingly falling short of the demands of modern agriculture. Their limitations include inefficiency and limited accuracy. They also encounter difficulties in promptly dealing with unforeseen health problems, such as calf diarrhea. Consequently, computer vision technology presents itself as a highly promising alternative solution [6].

Computer vision technology has advanced animal behavior recognition capabilities. It has also enhanced objectivity and continuity in recognizing pig and cow behaviors [7]. A systematic review by Rohan et al. [8] found that deep learning models recognize 13 types of livestock behavioral issues, covering 44 specific behaviors. Exploring technological frontiers, Wang et al. [9] focused on neural network models for sequential data. They investigated RNN models, including LSTM and GRU. In particular, their findings indicate that RNN models reduce computational and memory needs while maintaining high accuracy.

For calf behavior monitoring, various deep learning frameworks have been introduced to attain highly precise recognition of calf behaviors. Qiao et al. [10] proposed a framework integrating C3D and ConvLSTM networks. The framework achieved an accuracy of 90.32% in the recognition of calf behavior. Han et al. [11] emphasized the importance of recognizing abnormal behaviors for calf health. They introduced a two-branch temporal excitation and aggregation framework (DB-TEAF). Cirera et al. [12,13] focused on recognizing equine behavior time budgets from video. They analyzed activities such as feeding, resting, lying, and moving. They proposed a multi-input, multi-output method. This method outperformed single-input and single-output strategies.

However, when applying computer vision methods [14,15,16,17,18,19] to capture calf behavioral features, a critical challenge involves the efficiency of long-sequence feature extraction. This efficiency directly impacts the comprehensiveness and accuracy of behavior recognition. Despite the effectiveness of these methods, environmental factors pose significant obstacles. Complex conditions—such as obstacles, lighting variations, and overlapping occlusions—restrict accessible recognition features, thereby diminishing recognition accuracy. Thus, improving the efficiency of long-sequence feature extraction is imperative for achieving robust behavior recognition.

To improve long-sequence feature extraction efficiency, many researchers have proposed the Mamba architecture. Gu and Dao specifically introduced Mamba as a linear-time sequence modeling framework with selective state spaces [20]. This well-designed architecture, coupled with a selective SSM mechanism, overcomes prior limitations. Additionally, it enhances the model’s expressive power and enables more efficient management of input sequence information. By effectively leveraging SSM’s selection mechanism, Mamba extracts and prioritizes crucial sequence features for utilization. Crucially, it maintains linear time complexity. This enables efficient feature extraction and improved generation throughput. Mamba shows excellent performance across various tasks. Therefore, this method presents a promising architecture. Further research can explore its applications in animal behavior recognition.

The main contributions of this paper are as follows:

(1) In this paper, we propose a novel S_T_Mamba model designed to recognize Jinnan calf diarrhea behavior efficiently. This model can establish temporal dependencies by leveraging a sequence processing strategy. Additionally, it can construct spatial long-range feature relationships in images through the tree state space module.
(2) This paper initially introduces a sequence processing strategy, which builds temporal dependencies underlying the video. This strategy explores the temporal features of the diarrhea behavior of Jinnan calves. Subsequently, it can significantly improve the performance of calf diarrhea behavior recognition.
(3) This paper proposes an innovative tree state space module (TreeSSM). It can effectively capture long-range dependencies in the spatial domain of the image. Furthermore, TreeSSM enables the model to accurately characterize the subtle pattern differences underlying similar behaviors. Consequently, it will further enhance the discriminative ability of S_T_Mamba.
(4) This paper first establishes a median-scale dataset for Jinnan calf diarrhea behavior. This dataset consists of five classes of behavior, and each of them contains approximately 700 videos. Specifically, the duration of each video ranges from 5 to 10 s. This novel Jinnan calf diarrhea dataset is collected from a natural environment, which will provide a solid foundation for research in the field of calf diarrhea.

This paper is structured as follows: Section 1 introduces the significance of accurate diarrhea behavior recognition in Jinnan calves and the limitations of existing techniques, setting the stage for the proposed S_T_Mamba model; Section 2 reviews the development of animal behavior recognition methods from sensors to traditional CV and then to deep learning, and points out the shortcomings of current research in long-sequence efficiency and subtle behavior discrimination, thus leading to the S_T_Mamba model and the innovations proposed in this paper to solve these challenges; Section 3 presents the dataset collected from a natural farming environment and describes the architecture of the proposed S_T_Mamba model; Section 4 compares and analyzes the performance of the S_T_Mamba model against several benchmark models; Section 5 discusses the model’s limitations, explores potential factors affecting the accuracy of model in various farming environments, and outlines future research directions, including dataset expansion and model optimization; Section 6 emphasizes the state-of-the-art performance achieved by the S_T_Mamba model in Jinnan calf diarrhea behavior recognition and highlights its significance for improving calf health management.

## 2. Related Work

Wearable sensors, such as accelerometers embedded in collars or ear tags, have been extensively explored for animal behavior monitoring [2,3]. These methods typically employ machine learning algorithms like SVM [2] or LSTM [9] to classify behaviors based on time-series motion data. Their primary advantage lies in the ability to provide continuous, high-frequency data streams. However, their adoption is hampered by significant limitations: (1) high cost and intrusiveness, as the physical devices impose additional burdens on animals and require maintenance; (2) limited behavioral scope, often failing to effectively capture complex behaviors or subtle symptoms like diarrhea, which may not manifest pronounced unique motion patterns; (3) practical challenges in large-scale deployment, including battery life and data retrieval issues. These constraints have spurred the exploration of non-invasive, vision-based alternatives.

Prior to the deep learning era, traditional computer vision methods relied on hand-crafted features (e.g., HOG, SIFT) combined with classifiers like SVM for behavior recognition. These approaches focused on extracting manually designed visual cues from images or videos. While beneficial for their interpretability and lower computational demand, they suffer from critical drawbacks in complex farm environments: (1) Poor generalization and robustness: The hand-crafted features are often insufficient to cope with the vast variations in lighting, occlusion, posture, and background clutter encountered in real-world settings [7]. (2) Limited representational power: These features struggle to capture the high-level semantic information and subtle spatiotemporal dynamics necessary for distinguishing visually similar behaviors [7,8]. This inherent weakness paved the way for data-driven deep learning methods.

Deep learning has revolutionized video behavior recognition. Convolutional Neural Networks (CNNs) [21] became the established standard for spatial feature extraction. To model temporal information, architectures like C3D, Two-Stream Networks, and ConvLSTM [10] were developed. Vision Transformers (ViTs) [22] and Pyramid Vision Transformers (PVTs) [23] have shown strong performance by leveraging self-attention mechanisms to capture global contexts. Most recently, state space models (SSMs), particularly the Mamba architecture [20,24], have emerged as a promising alternative for long-sequence modeling due to their linear time complexity and effective dependency capture. However, two pivotal challenges persist: (1) Long-sequence modeling efficiency: While effective, many existing models suffer from quadratic computational complexity or memory constraints, limiting their efficiency in processing long video sequences crucial for understanding behavioral context. (2) Discrimination of subtle inter-class differences: Despite their power, capturing fine-grained spatial details and long-range pixel dependencies necessary to differentiate behaviors with high visual similarity remains a challenge. Our proposed S_T_Mamba model, integrating a sequence processing strategy and a novel TreeSSM, is designed specifically to address these two limitations.

In summary, despite the remarkable progress of existing research, two key challenges still remain: (1) how to efficiently model spatiotemporal dependencies in long video sequences; (2) how to accurately capture subtle differences between similar behaviors (e.g., walking versus standing). The recent Mamba architecture has provided new ideas for efficient sequence modeling, but its application to vision tasks is still in its early stages, and its inherent temporal invariance assumption limits its ability to model long-distance dependencies in the spatial domain. To this end, this study proposes the S_T_Mamba model to address the above challenges. Our main innovations are (1) introducing sequence processing strategies to explicitly model the temporal dynamics of behaviors; (2) proposing a tree state space module (TreeSSM) to adaptively capture long-range spatial dependencies by constructing a minimum spanning tree of image pixels, thereby enhancing the ability of the model to distinguish subtle behavior patterns.

## 3. Materials and Methods

This section provides a detailed description of the materials and methods employed in this study. We first introduce the dataset collected from a natural farming environment and then provide a comprehensive explanation of the proposed S_T_Mamba model architecture.

### 3.1. Dataset

The dataset utilized in this paper to recognize Jinnan calf diarrhea behavior was collected from a farm located in Yongji, Shanxi Province. The farm is 100 m long and 24 m wide, containing approximately 45 Jinnan calves. These calves were up to six months old. To achieve comprehensive real-time monitoring of Jinnan calf diarrhea behavior, cameras were installed 2.5 m above the four corners of each pen. Four cameras were deployed at 45°, 135°, −45°, and −135°. To obtain the recognition dataset of Jinnan calf diarrhea behavior, a total of 3606 videos of Jinnan calf diarrhea behavior were collected over a one-month period. The video collection period spanned from 0:00 to 24:00 across diverse weather conditions (sunny/cloudy/rainy = 6:3:1), capturing comprehensive variations including different postures, orientations, lighting conditions, and backgrounds. Subsequently, each video was evenly divided at a rate of 25 frames per second, and the strategy of random frame extraction was adopted to construct the dataset. The dataset covers five different diarrhea-related behaviors of Jinnan calves, including eating, walking, standing, lying, and diarrhea. The behavior categories along with their corresponding descriptions and labels in the dataset are presented in Table 1.

**Figure 1:**
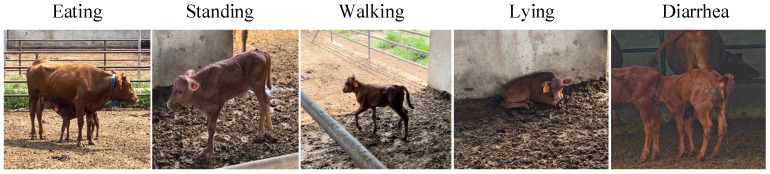
Samples of several Jinnan calf diarrhea behaviors.

**Figure 2:**
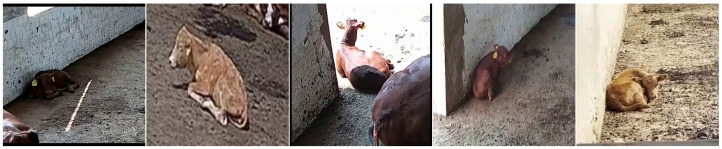
A visual comparison of the lying behavior exhibited by different calves under varying conditions.

Regarding the number of video samples for these five behavioral categories, the number of samples for the eating behavior is 722, for the standing behavior is 727, for the walking behavior is 730, for the lying behavior is 727, and for the diarrhea behavior is 700. The detailed distribution of video samples across these five behavioral categories is summarized in Table 2. The dataset was randomly split into a training set and a test set in an 8:2 ratio. Specifically, the training set comprised 44,772 images, and the test set contained 9594 images. All images were uniformly resized to 224 × 224 pixels. Figure 1 presents several samples from the Jinnan calf diarrhea behavior recognition dataset. Figure 2 provides a visual comparison of the same behavior (such as lying) exhibited by different calves under different conditions.

### 3.2. S_T_Mamba

**Figure 3:**
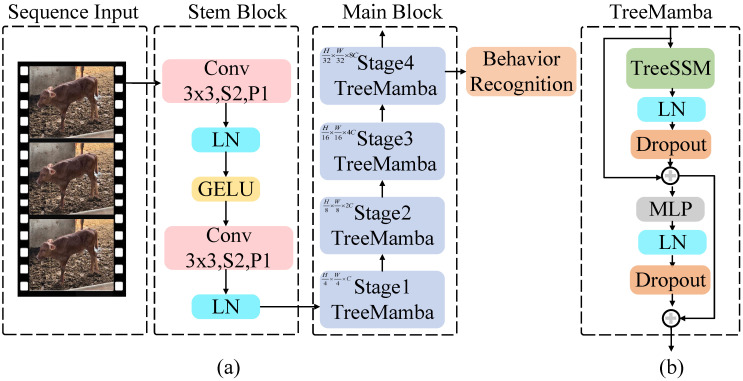
The diagram of the S_T_Mamba model. (**a**) represents the overall architecture of the S_T_Mamba model, while (**b**) denotes the detailed structure of the TreeMamba block.

To enhance the performance of Jinnan calf diarrhea behavior recognition, we developed an efficient model called S_T_Mamba, as illustrated in Figure 3. It mainly consists of an input component that employs a sequence processing strategy, a stem block, and several TreeMamba blocks. The core module of TreeMamba is a tree state space module.

#### 3.2.1. Sequence Processing Strategy

The sequence processing strategy takes several sampled frames from the corresponding video as inputs. By utilizing the sequence processing strategy, the model can better understand changes in calf diarrhea behaviors over time. Concretely, this design enables the model to adaptively select temporal information and organize it into sequence, further improving its ability to extract key temporal features of calf diarrhea behaviors.

#### 3.2.2. Stem Block

The stem block comprises two convolutions, each followed by a Layer Normalization (LN) layer. Additionally, after the first convolution, there is a GELU activation function.

#### 3.2.3. TreeMamba

The S_T_Mamba model employs a hierarchical network structure that processes the input feature map in multiple stages. Each processing stage includes TreeSSM and MLP. Additionally, LayerNorm and dropout operations are applied after each module, and a residual connection is leveraged between each module.

#### 3.2.4. TreeSSM

**Figure 4:**
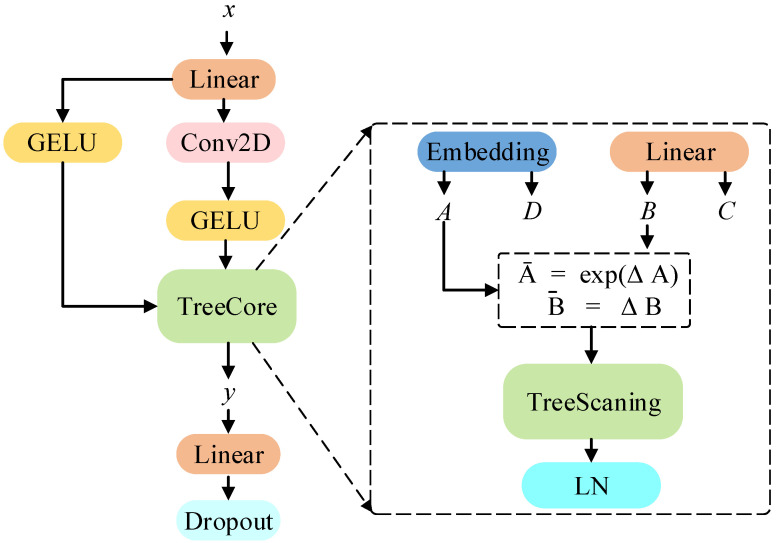
An overview of TreeSSM.

TreeSSM is an extended variant of SSM [20]. The core insight of TreeSSM is a modified state space tree selection mechanism, which makes the S_T_Mamba model perform well in capturing long-range spatial dependencies. In addition, TreeSSM proposes a TreeScanning algorithm that constructs a connectivity map of pixel points in the image. The detailed architecture of TreeSSM is shown in Figure 4. The specific implementation procedure of TreeSSM is systematically presented in Algorithm 1. In this subsection, we will first explain the basic SSM and then TreeSSM.
**Algorithm 1** TreeSSM process.
     **Input:** Input features *x*, parameters {A,B,C,D}, Δ
     **Output:** Transformed features *y*
  1:  **// Feature projection and discretization**
  2:  $\Delta \leftarrow \mathrm{softplus}(\mathrm{Linear}\left(x\right)+{\mathrm{bias}}_{\Delta })$
  3:  A←exp(Δ∗A)
  4:  B←Δ∗B
  5:  **// Minimum spanning tree construction**
  6:  features_2d←reshape(x,(H,W))
  7:  adj_matrix←cosine_similarity(features_2d)
  8:  mst←boruvka_algorithm(adj_matrix)
  9:  **// Tree traversal ordering**
10:  sorted_order,parent,child←BFS(mst,root=random_node)
11:  **// Tree-structured feature refinement**
12:  aggregated_features←tree_scan_refine(B∗x,A,sorted_order,parent,child)
13:  **// Output computation**
14:  y←LayerNorm(aggregated_features)∗C+D∗x
15:  **return** *y*


**SSM**: The state space model is typically regarded as a continuous linear time-invariant system. This system maps input feature $x\in R^{N\times F}$ through a state vector $h^{\prime }\left(t\right)\in R^{N\times F}$ to output feature $y\in R^{N\times F}$, where *N* represents the size of the hidden state, *F* denotes the channel number of the signal, and *t* indicates the time step. Specifically, it can be formulated as the following Equation (1):

(1) $$h^{\prime }\left(t\right)=Ah\left(t\right)+Bx\left(t\right)\;,\;y\left(t\right)=Ch\left(t\right)+Dx\left(t\right)$$

where A, B, C, and D are continuous variables, A$\;\in R^{1\times F}$, B$\;\in R^{N\times 1}$, C$\;\in R^{N\times 1}$, and D$\;\in R^{1\times F}$.

In order to effectively apply SSM to process specific tasks, it needs to be discretized. Concretely, the zero-order hold method is used to discretize a continuous system. It discretizes the continuous variables (A, B, C, D) at a specified sampling time, converting them to the corresponding discrete parameters ($\bar{A}$, $\bar{B}$, $\bar{C}$, $\bar{D}$) and the specified sampling time scale $\Delta \in R^{N\times F}$. In addition, many improved methods [20] use an approximation of $\bar{B}$ based on the first-order Taylor Series. The specific discrete process is expressed in Equation (2).

(2) $$\bar{A}=e^{\Delta A}\;,\bar{B}=\Delta B\;,\bar{C}=C\;,\bar{D}=D\;$$

where $\bar{A}$ $\in R^{N\times F}$, $\bar{B}$ $\in R^{N\times F}$, $\bar{C}$ $\in R^{N\times 1}$, and $\bar{D}$ $\in R^{1\times F}$.

**TreeSSM**: Although SSM has several remarkable advantages, such as its promising linear-time reasoning capacity, parallelized training, and robust performance on long-context tasks, it suffers from the limitation of inherent time invariance, which constrains its effectiveness. To address this problem, TreeSSM integrates a dynamic mechanism to select input features into the sequence. The process of TreeSSM is expressed in Equations (3)–(5). Specifically, the hidden state $h_{i}$ is derived by Equation (3), and the output $y_{i}$ is obtained based on $h_{i}$ by Equation (5).

(3) $$h_{i}=\sum_{j\in V}W\left(E_{ij}\right){\bar{B}}_{i}x_{i}$$

where i∈{1,2,…,N}, with *i* representing a vertex; *V* denotes the set of vertices; ${\bar{B}}_{i}\in R^{N\times F}$ and ${\bar{B}}_{i}$ indicates the input matrix; $x_{i}\in R^{1\times F}$, where $x_{i}$ represents the feature of the input vertex; $E_{ij}$ is the edge connecting the *i*-th vertex to the *j*-th vertex; and $W\left(E_{ij}\right)\in R^{N\times F}$, with $W(E_{ij})$ representing the weight of edge $E_{ij}$. This is presented in Equation (3).

(4) $$W\left(E_{ij}\right)=\prod_{v\in N_{ij}}\bar{A}\left(v_{i},v_{j}\right)=\prod_{v\in N_{ij}}\frac{F\left(v_{i}\right)⊙F\left(v_{j}\right)}{∥F\left(v_{i}\right)∥∥F(v_{j})∥}$$

where $N_{ij}$ is the index set of all vertices on edge $E_{ij}$, it takes *i* as the root node, and all the children nodes of *i* are in the set *j*. $\bar{A}\left(v_{i},v_{j}\right)$ denotes the transition matrix, which is shown by Equation (4). ⊙ denotes the dot product of vertices $v_{i}$ and $v_{j}$, $F(v_{i})$ and $F(v_{j})$ indicate the features of vertices $v_{i}$ and $v_{j}$, and ∥∥·∥∥ represents the norm of features.

(5) $$y_{i}=Norm\left(h_{i}\right){\bar{C}}_{i}+{\bar{D}}_{i}x_{i}$$

where the Norm stands for the LayerNorm operation; ${\bar{C}}_{i}\in R^{N\times 1}$, and ${\bar{C}}_{i}$ represents the output matrix; and ${\bar{D}}_{i}\in R^{1\times F}$, with ${\bar{D}}_{i}$ denoting the command coefficient.

The following further explains the process of TreeSSM in detail. Specifically, given an input feature $x_{i}$, TreeSSM constructs an undirected *n*-connected graph, denoted as G=(V,E). Here, *n* is a hyperparameter that indicates the number of neighboring nodes connected to each pixel, which is set to 4. *V* represents the set of vertices, which in this task also corresponds to the set of pixel embeddings, and *E* signifies the edges of the graph.

To eliminate edges exhibiting significant differences, TreeSSM employs the Boruvka algorithm [25], yielding a minimum spanning tree. Additionally, the weights of these edges in the minimum spanning tree are determined by measuring the feature similarity between adjacent vertices, which is calculated using the cosine distance.

Specifically, the TreeSSM process involves an iterative traversal of each vertex in the minimum spanning tree. Treating each vertex as the root, it aggregates the features of the four sub-vertices. The aggregation process incorporates the weights of the edges to produce a transformed state. When applied within this geometric configuration, it prioritizes interactions between vertices that exhibit small spatial and feature distances.

The output feature $y_{i}$ is obtained by applying the LayerNorm operation to the hidden state $h_{i}$ of the *i*-th vertex. This normalized hidden state is then multiplied by ${\bar{C}}_{i}$ and added to ${\bar{D}}_{i}$. In summary, the TreeSSM process constructs a minimum spanning tree, aggregates the features based on the edge weights and the transition matrices, and finally normalizes the hidden states to produce the output feature.

### 3.3. The Loss Function

In the calf diarrhea behavior recognition task, the popular cross-entropy loss function used for recognition tasks is utilized, and it is defined as Equation (6):

(6) $$\mathrm{Loss}=-\sum_{n=1}^{N}\sum_{k=1}^{K}p_{n}^{k}\log\left({\hat{p}}_{n}^{k}\right)$$

where *N* represents the number of samples, *K* denotes the number of categories, and $p_{n}^{k}$/${\hat{p}}_{n}^{k}$ indicate the real/predicted label for the corresponding sample.

### 3.4. Model Configuration

The detailed configuration of the proposed S_T_Mamba model is summarized in Table 3. To ensure a fair comparison, all models were trained and evaluated under identical hyperparameter settings. The architecture of comparative models (e.g., S_ConvNeXt, S_ViT) follows their standard baseline designs while integrating our sequence processing strategy.

## 4. Experiments and Analysis

This section presents the experimental results and analysis of the proposed S_T_Mamba model. We commence by detailing the experimental setup, subsequently presenting a comprehensive performance comparison between our model and several benchmark models.

### 4.1. Experimental Setup

All experiments in this study were carried out on an Ubuntu 18.04 hardware system. The experimental parameters were set as follows: the batch size was 32, the initial learning rate was 0.001, and the total number of training rounds was 250 epochs. Additionally, the Adam optimizer was employed during the training process.

### 4.2. Evaluation of the Effectiveness of Different Models

In order to verify the effectiveness of our proposed model, S_T_Mamba, we compared it with several popular models, including ConvNeXt [21], ViT [22], PVT [23], Bi_Mamba [26], and Mamba [24]. Since these experiments involved a sequence processing strategy, we renamed the model names to S_ConvNeXt, S_ViT, S_PVT, S_Bi_Mamba, and S_Mamba for clarity.

**Figure 5:**
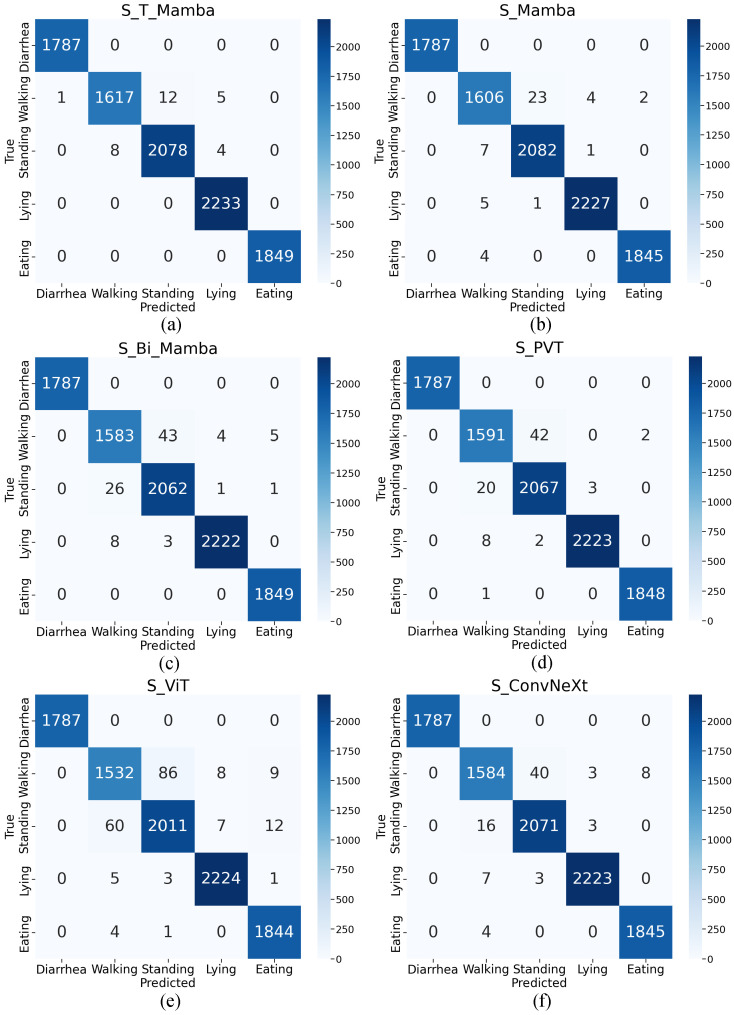
The confusion matrices of different models for calf diarrhea behavior recognition. Each subfigure represents a distinct model: (**a**) S_T_Mamba, (**b**) S_Mamba, (**c**) S_Bi_Mamba, (**d**) S_PVT, (**e**) S_ViT, and (**f**) S_ConvNeXt. The matrices illustrate the true versus predicted behaviors (Diarrhea, Walking, Standing, Lying, Eating), with diagonal elements indicating correct predictions and off-diagonal elements showing misclassifications.

To verify the calf diarrhea behavior recognition performance, the confusion matrix was utilized as a visualization tool to clearly illustrate the mapping relationship between the predicted and the actual behaviors. Figure 5 demonstrates that the S_T_Mamba model achieves superior performance in the calf diarrhea behavior recognition task. Specifically, the proposed model correctly classifies a relatively high number of samples for each behavior category, thereby validating the effectiveness of the S_T_Mamba model.

The comparison results are presented in Table 4. Table 4 demonstrates that our proposed S_T_Mamba model outperforms all other models. Specifically, the S_T_Mamba model achieves 99.78% accuracy, which is 0.64%, 1.99%, 0.59%, 0.71%, and 0.25% higher than those of S_ConvNeXt, S_ViT, S_PVT, S_Bi_Mamba, and S_Mamba, respectively. Additionally, the loss value of S_T_Mamba is 1.658, which is lower by 0.152, 0.239, 0.172, 0.158, and 0.109 compared to those of S_ConvNeXt, S_ViT, S_PVT, S_Bi_Mamba, and S_Mamba, respectively. In terms of Precision, Recall, and F1-Score, S_T_Mamba achieved 99.68%, 99.66%, and 99.67%, respectively, which were 0.17% to 1.80%, 0.19% to 1.85%, and 0.18% to 1.83% higher than the other models, indicating that it has a significant advantage in calf diarrhea behavior recognition tasks. While the proposed S_T_Mamba architecture achieves state-of-the-art accuracy, it entails higher computational costs (29.96 M Params, 4.78 G FLOPs) and notably higher inference latency, primarily due to the iterative tree construction and aggregation process of the novel TreeSSM module.

**Figure 6:**
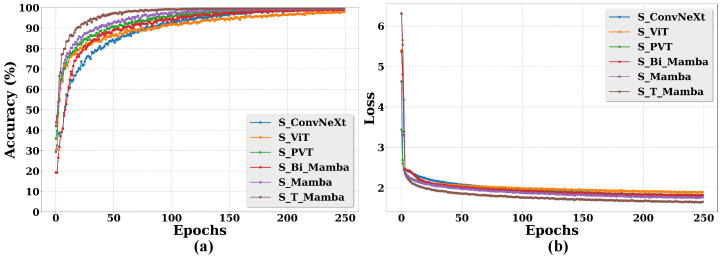
Comparative performance of various models for calf diarrhea behavior recognition through accuracy and loss curves across different epochs. (**a**) represents the accuracy and (**b**) denotes the loss.

To further validate the effectiveness of S_T_Mamba, Figure 6 illustrates both the accuracy and loss curves of all the compared models across various epochs.

Figure 6a demonstrates that the accuracy of S_T_Mamba consistently surpasses that of the S_ConvNeXt, S_ViT, S_PVT, S_Bi_Mamba, and S_Mamba models. Furthermore, as shown in Figure 6b, the S_T_Mamba model attains the lowest loss value. These results further validate the effectiveness of S_T_Mamba.

Table 5 demonstrates the superior performance of the S_T_Mamba model in the calf diarrhea behavior recognition task. Specifically, the model achieved the highest accuracy in recognizing the three behaviors of “Diarrhea,” “Lying,” and “Eating.” For the recognition of “Walking” and “Standing” behaviors, although its results were slightly inferior to those of the former three, it still reached a suboptimal level. Overall, the S_T_Mamba model has yielded ideal results in calf diarrhea behavior recognition, strongly validating its effective performance in the corresponding recognition tasks.

**Figure 7:**
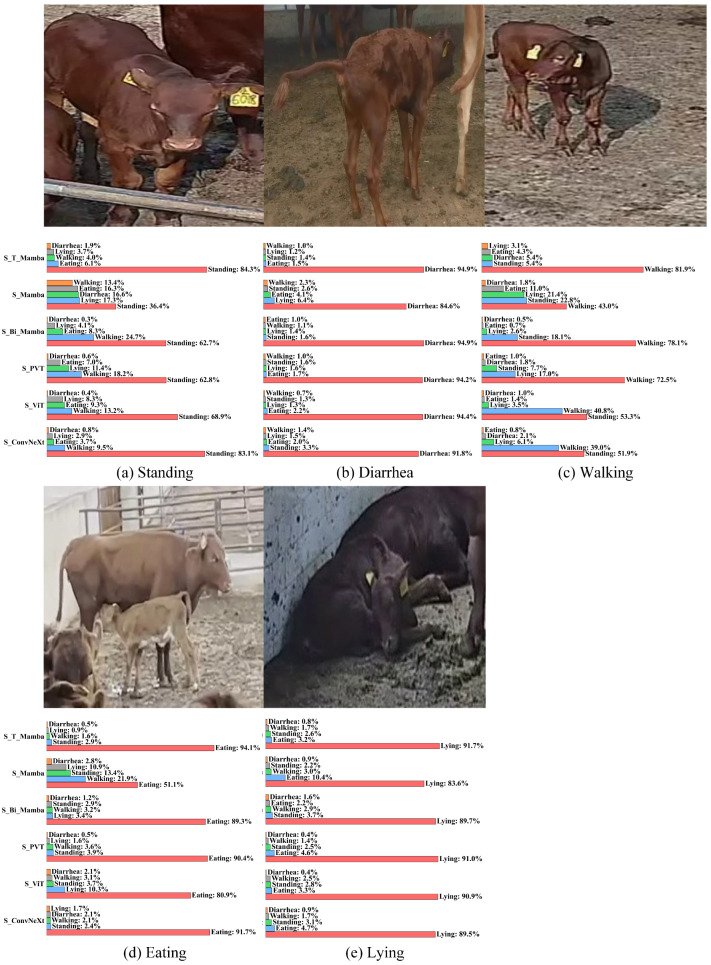
A comparative visualization of various models for calf diarrhea behavior recognition. The recognition accuracy of each model for different behaviors is presented: (**a**) Standing, (**b**) Diarrhea, (**c**) Walking, (**d**) Eating, (**e**) Lying. The red bars highlight the accuracy of each model for the specific behavior depicted in each respective subfigure.

As shown in Figure 7, it presents the recognition results of various models for different behaviors. For the standing behavior depicted in Figure 7a, compared to the accuracies of the S_Mamba, S_Bi_Mamba, S_PVT, S_ViT, and S_ConvNeXt models, which are 36.4%, 62.7%, 62.8%, 68.9%, and 83.1%, respectively, our model demonstrates superior performance, achieving an accuracy of 84.3%. Additionally, as illustrated in Figure 7b,d,e, for the behaviors of diarrhea, eating, and lying, our method attains accuracies of 94.9%, 94.1%, and 91.7% respectively. Finally, regarding the “Walking” behavior, as shown in Figure 7c, due to the high visual similarity between the “Walking” and “Standing” behaviors, recognizing the “Walking” behavior presents a significant challenge. Our proposed S_T_Mamba model effectively addresses this challenge and achieves a high accuracy of 81.9% in correctly identifying the “Walking” behavior. In contrast, the S_ViT model achieves accuracies of 40.8% for “Walking” and 53.3% for “Standing”, while the S_ConvNeXt model attains accuracies of 39.0% for “Walking” and 51.9% for “Standing”. This indicates that S_ViT and S_ConvNeXt experience relatively serious confusion issues during the recognition process. Based on the above analysis, the superior performance of the S_T_Mamba model in accurately recognizing calf diarrhea behaviors has been validated.

The reasons for the superiority of the S_T_Mamba model in recognizing calf diarrhea behavior can be summarized as follows. Firstly, the sequence processing strategy employs sequence data as input to better capture the temporal relationships within the video. Secondly, the tree state space module significantly enhances the effectiveness of recognizing calf diarrhea behavior by thoroughly extracting and integrating long-range pixel correlation features from video frames, thereby enabling the precise distinction of subtle pattern variations among similar behaviors. Consequently, the effective modeling of temporal dependencies and comprehensive long-range feature extraction greatly enhance the performance of calf diarrhea behavior recognition.

### 4.3. Evaluation of the Effectiveness of Sequence Processing Strategy

This subsection focuses on validating the impact of the sequence processing strategy for calf diarrhea behavior recognition. Concretely, several benchmark models, including ConvNeXt [21], ViT [22], PVT [23], Bi_Mamba [26], and T_Mamba, with or without sequence processing, are employed for this validation. The comparison results are presented in Table 6. To clarify, in Table 6, ConvNeXt [21], ViT [22], PVT [23], Bi_Mamba [26], and T_Mamba represent those models without sequence processing, while S_ConvNeXt, S_ViT, S_PVT, S_Bi_Mamba, and S_T_Mamba represent the corresponding models with sequence processing.

Table 6 shows that the models with sequence processing perform better than those without sequence processing. Specifically, S_ConvNeXt achieves 99.15% accuracy, marking a significant 56.22% improvement compared to ConvNeXt. The accuracy of S_ViT is 97.83%, which is 26.31% higher than that of ViT. The accuracy of S_PVT is 99.19%, surpassing PVT by 26.70%. S_Bi_Mamba achieves an accuracy of 99.08%, which is 48.30% higher than that of Bi_Mamba. S_T_Mamba achieves 99.78% accuracy, representing an increase of 14.33% compared to T_Mamba.

**Figure 8:**
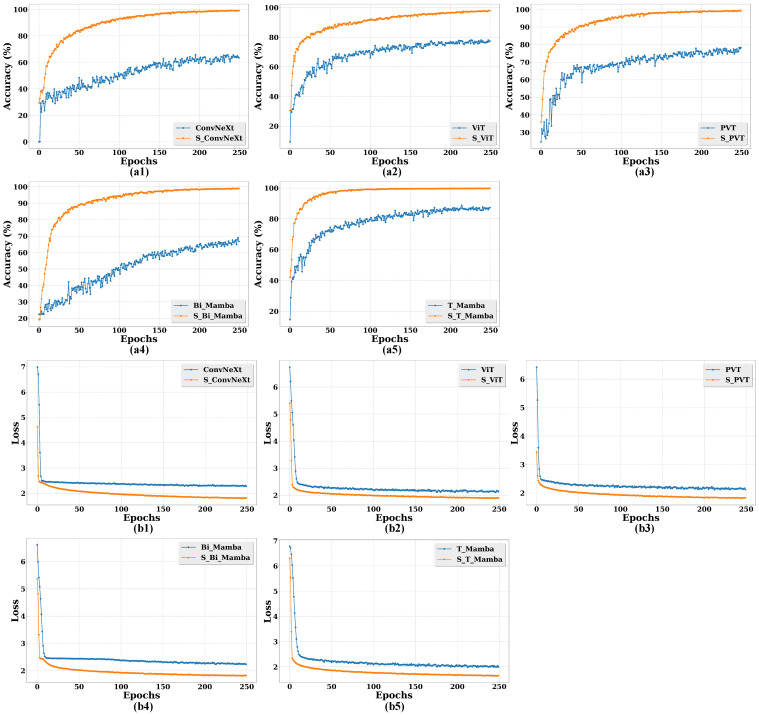
Comparative performance of different models with and without sequence processing strategy for calf diarrhea behavior recognition evaluated through accuracy and loss curves across different epochs. Specifically, model names with “S” indicate the inclusion of a sequence processing strategy, while those without “S” indicate its absence. (**a1**–**a5**): Accuracy curves for different model comparisons. (**b1**–**b5**): Loss curves for corresponding model comparisons.

Moreover, to further demonstrate the effectiveness of the sequence processing strategy in those models, we compare the accuracy and loss curves of different models with and without a sequence processing strategy in Figure 8. Specifically, it can be seen from Figure 8(a1–a5) that those models leveraging a sequence processing strategy exhibit a significant improvement in accuracy compared to those models without sequence processing. In Figure 8(b1–b5), the loss values are also minimized in those models with sequence processing.

The reasons why models with sequential input achieve superior accuracy in calf diarrhea behavior recognition can be summarized as follows. Firstly, the sequential input enables these models to capture more temporal information from the video, thereby facilitating the extraction of key discriminative features that are indicative of calf diarrhea behavior. Consequently, models with sequential input demonstrate enhanced performance in recognizing calf diarrhea behavior.

### 4.4. Evaluation of the Effectiveness of Different Sequence Frame Lengths

In order to evaluate the impact of different sequence frame lengths on calf diarrhea behavior recognition, several benchmark models with different sequence frame lengths are leveraged for comparison. Specifically, sequence lengths of 5, 10, 15, or 20 frames per video are chosen here. Additionally, comparison models include S_ConvNeXt, S_ViT, S_PVT, S_Bi_Mamba, and S_T_Mamba. Detailed results are reported in Table 7.

Table 7 demonstrates that those models with sequence frame lengths of 20 outperform those with sequence frame lengths of 5, 10, and 15. Specifically, the S_ConvNeXt model with a sequence frame length of 20 achieves 99.15% accuracy, which is 17.41%, 8.40%, and 2.25% higher than the models with sequence frame lengths of 5, 10, and 15, respectively. The accuracy of the S_ViT model with a sequence frame length of 20 is 97.83%, which shows an improvement of 11.18%, 4.97%, and 2.03% compared to the models with sequence frame lengths of 5, 10, and 15 separately. The accuracy of the S_PVT model with a sequence frame length of 20 is 99.19%, exceeding the models with sequence frame lengths of 5, 10, and 15 by 7.05%, 2.48%, and 0.75%. When the sequence frame length is 20, the accuracy of the S_Bi_Mamba model is 99.08%, which is 11.03%, 3.04%, and 1.18% better than that of the sequence frame lengths of 5, 10, and 15, correspondingly. The recognition accuracy of S_T_Mamba is 99.78% when the sequence frame length is 20, marking 3.05%, 0.47%, and 0.03% improvement compared to sequence frame lengths of 5, 10, and 15.

**Figure 9:**
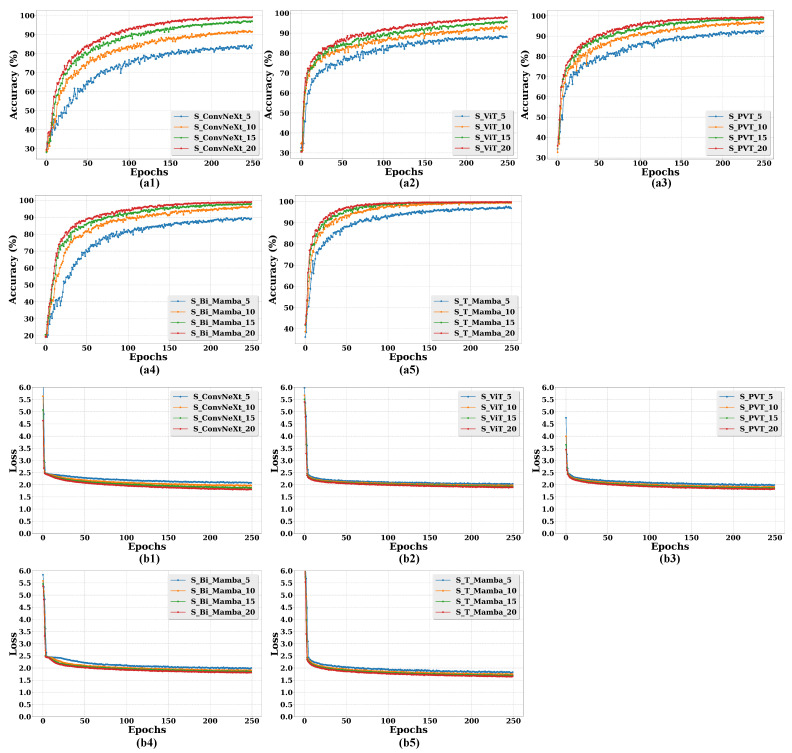
Comparative performance of different models with different sequence frame lengths for calf diarrhea behavior recognition evaluated through accuracy and loss curves across different epochs. Here, 5, 10, 15, and 20 represent the sequence frame lengths. (**a1**–**a5**): Accuracy curves for different sequence frame length comparisons. (**b1**–**b5**): Loss curves for corresponding sequence frame length comparisons.

Furthermore, to further validate the effectiveness of the different sequence frame lengths in those models, we compare the accuracy and loss curves of several different models with different sequence frame lengths in Figure 9. As shown in Figure 9(a1–a5), models employing a sequence frame length of 20 present a uniform enhancement in accuracy compared to those models with sequence frame lengths of 5, 10, and 15. In Figure 9(b1–b5), models with a sequence frame length of 20 achieve the lowest loss values.

Models with a sequence frame length of 20 demonstrate outstanding performance in calf diarrhea behavior recognition compared to those with sequence frame lengths of 5, 10, and 15. The reason for this is that the longer the sequence frame length, the better these models represent the changes in calf diarrhea behavior, and the more effectively they capture the temporal features underlying the video. As a result, they achieve promising performance in calf diarrhea behavior recognition.

### 4.5. Evaluation of the Effectiveness of Different Sampling Strategies

In order to assess the performance of different sampling strategies, a comparative analysis was conducted between random and uniform sampling strategies. The results of these comparative assessments are demonstrated in Table 8.

Specifically, the recognition accuracy of S_ConvNeXt, which employed random sampling, reached 99.15%, marking a 0.47% improvement over uniform sampling. S_ViT achieves a recognition accuracy of 97.83% when employing the random sampling strategy, outperforming the uniform sampling strategy by 0.22%. Similarly, S_PVT demonstrates a recognition accuracy of 99.19% with random sampling, exceeding the uniform sampling strategy by 0.28%. S_Bi_Mamba, when utilizing random sampling, attains a recognition accuracy of 99.08%, marking a 0.65% improvement compared to uniform sampling. Lastly, S_T_Mamba, under random sampling conditions, achieves an impressive recognition accuracy of 99.78%, surpassing the uniform sampling strategy by 0.04%. In summary, across all models, the random sampling strategy consistently yields a slight improvement in recognition accuracy over the uniform sampling strategy, varying from 0.04% to 0.65%.

The random sampling strategy enhances the performance of different models. The uniform sampling strategy may cause these models to focus excessively on fixed regions and ignore other significant information in the video. In contrast, the random sampling strategy provides a richer set of temporal features for these models. It allows these models to learn from any point in the video, thereby capturing key information in the dataset more comprehensively.

### 4.6. Evaluation of the Effectiveness of TreeSSM

In order to evaluate the effectiveness of TreeSSM in calf diarrhea behavior recognition, a comparative analysis was conducted between models with and without TreeSSM.

Specifically, we compared the performance of two models: S_Mamba and S_T_Mamba. The S_Mamba model does not incorporate the TreeSSM component, whereas the S_T_Mamba model integrates TreeSSM. The performance metrics used for comparison are accuracy (%) and loss.

The results show that the model with TreeSSM performs better than that without TreeSSM. The S_T_Mamba model achieves an accuracy of 99.78%, which is 0.25% higher than that of the S_Mamba model (99.53%). Moreover, the loss value of the S_T_Mamba model is 1.658, which is 0.109 lower than that of the S_Mamba model (1.767). These results clearly demonstrate the positive impact of incorporating TreeSSM on the model’s performance in calf diarrhea behavior recognition.

**Figure 10:**
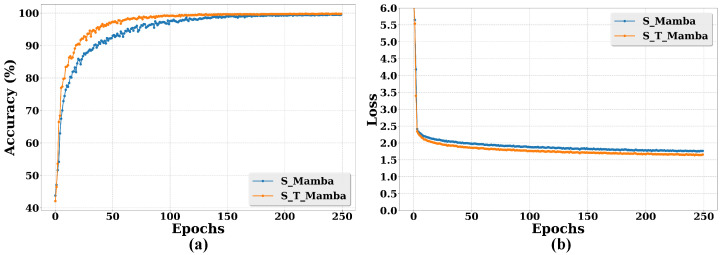
Comparative performance of a model with and without TreeSSM for calf diarrhea behavior recognition through accuracy and loss curves across different epochs. Specifically, S_T_Mamba represents the model with TreeSSM, while S_Mamba represents the model without TreeSSM. (**a**) represents the accuracy and (**b**) denotes the loss.

To further verify the performance of TreeSSM, Figure 10 shows the accuracy and loss curves of these models for different epochs. In Figure 10a, the accuracy of S_T_Mamba consistently surpasses that of the S_Mamba model. Furthermore, as shown in Figure 10b, the S_T_Mamba model achieves the lower loss value. These results validate the effectiveness of TreeSSM.

S_T_Mamba demonstrates better performance than S_Mamba. The reasons for this improvement can be attributed to the fact that TreeSSM in S_T_Mamba constructs a minimum spanning tree on a connected graph based on the differences between neighboring features in the image. This process adaptively encodes the spatial information of the image into the tree structure. Subsequently, the model iteratively traverses each pixel by utilizing a state transfer mechanism, and it aggregates long-range pixel-associated features in the image. Thus, the TreeSSM module allows the model to effectively extract long-range features in the image, ultimately leading to better performance in calf diarrhea behavior recognition.

## 5. Discussion

In this research, the S_T_Mamba model achieved significant advances in the Jinnan calf diarrhea behavior recognition task. By optimizing the structure of the model, the S_T_Mamba model achieved significant advances, as evidenced by its state-of-the-art performance (99.78% accuracy) and lowest loss value (1.658) in the comparative experiments (see Table 4 and Figure 6), exhibiting strong adaptability and high accuracy in complex environments. This model provides livestock breeders with an efficient tool for real-time monitoring of calf health status.

The design concept of the S_T_Mamba model is mainly reflected in the following aspects. First, the model integrates a sequence processing strategy. This strategy is crucial for establishing temporal dependencies, which is confirmed by the substantial performance gains observed in models incorporating this strategy (see Table 6 and Figure 8).

By leveraging this strategy, the model can better explore the temporal features of calf behavior, thereby significantly improving the performance of calf diarrhea behavior recognition. Second, the innovative tree state space module (TreeSSM) is introduced. The ablation study (Section 4.6, Figure 10) clearly demonstrates its positive impact, where the integration of TreeSSM contributed to a 0.25% accuracy improvement and reduced loss by enabling the model to capture long-range spatial dependencies and distinguish subtle patterns. It can capture long-range dependencies in the spatial domain of images, enabling the model to accurately characterize the subtle pattern differences underlying similar behaviors. This further enhances the discriminative capacity of the S_T_Mamba model.

While the S_T_Mamba model demonstrates state-of-the-art performance in Jinnan calf diarrhea behavior recognition, this study has several limitations. First, the dataset scale remains moderate, which may limit generalization across diverse conditions. Second, environmental factors such as extreme lighting or occlusions were not fully evaluated. Third, the evaluation protocol employed a single, fixed train–test split. While this provides a clear benchmark, it does not incorporate statistical measures such as K-fold cross-validation or multiple runs with different random seeds to report confidence intervals. This choice was primarily pragmatic, balancing computational costs associated with processing long video sequences against the desire for extensive statistical validation. Fourth, subtle distinctions between similar behaviors require further refinement. Future work will focus on expanding the dataset, incorporating more robust statistical validation protocols, optimizing model efficiency, and integrating multi-modal sensors to enhance robustness.

This study adopted a classification retention validation strategy instead of K-fold cross-validation. Considering the actual research context and resource constraints, we believe that this method, combined with the independent operation of multiple different random seeds, can provide reliable and stable estimates for model performance. The balance of our dataset, the representativeness of the test set ensured through categoristic sampling, and its sufficiently large scale all enhance the robustness of the evaluation. We acknowledge the limitation of not using K-fold cross-validation, which could have provided a more comprehensive assessment of generalization ability across different data partitions. Despite this, we believe that the results obtained by the current strategy are reliable and of practical significance. Future research can adopt K-fold cross-validation to further verify and enhance the generalization ability of the proposed model.

Although this study has achieved remarkable results in algorithm accuracy, a key direction for the future will be a comprehensive assessment of its deployment feasibility in actual farm environments. This includes, but is not limited to, systematically evaluating the inference speed, power consumption and throughput of the model on embedded systems (such as Jetson AGX Orin, Nano) and GPU servers, and exploring lightweight technologies such as model pruning and quantization to meet the demanding resource constraints of edge devices while maintaining performance.

Furthermore, we aim to enhance data diversity by incorporating multi-farm environments and challenging conditions to improve model robustness. Real-time performance will be optimized through model quantization or pruning to meet on-farm deployment requirements. Finally, acoustic and environmental sensors will be integrated to enrich behavioral context understanding, thereby addressing limitations in dataset scale and environmental adaptability.

## 6. Conclusions

This paper proposes a novel behavior recognition model for Jinnan calf diarrhea, termed S_T_Mamba (Sequence Tree Mamba), which integrates a sequence processing strategy and a tree state space module. The proposed model effectively captures temporal features within an image by employing the sequence processing strategy. Furthermore, the tree state space module enhances the extraction of discriminative features from similar behaviors, enabling more accurate differentiation. Therefore, S_T_Mamba significantly improves Jinnan calf diarrhea behavior recognition performance. Additionally, a novel dataset for Jinnan calf diarrhea behavior recognition is first established in this paper. Experimental results demonstrate the effectiveness of the proposed model. Notably, S_T_Mamba achieves state-of-the-art performance in Jinnan calf diarrhea behavior recognition. Specifically, the model attains an accuracy of 99.78%, surpassing existing popular models by a margin of 0.59% to 1.99%.

## Figures and Tables

**Table 1:** Characteristics of the behavior categories in the dataset.

Behavior Category	Behavioral Description	Label
Eating	Suckled at the teat	Eating
Standing	Leg upright to support body	Standing
Walking	Alternated bending of limbs, trunk horizontal, head raised	Walking
Lying	Abdominal contact with ground	Lying
Diarrhea	Diluted excreta discharged from their tails	Diarrhea

**Table 2:** Number of video samples for five behavioral categories in Jinnan calves.

Behavior Category	Total Number of Videos	Number of Distinct Calves
Diarrhea	700	9
Eating	722	10
Standing	727	9
Walking	730	9
Lying	727	8
Total	3606	45

**Table 3:** Model configuration details.

Hyperparameter/Component	Value/Description
Input size	224×224×3
Sequence length	20
Batch size	32
Optimizer	Adam ($\beta_{1}=0.9$, $\beta_{2}=0.999$)
Initial learning rate	0.001
Learning rate scheduler	StepLR(0.1)
Training epochs	250
Weight decay	0.05
Stem block	2×Conv2D,GELU,LayerNorm
TreeMamba layers	4
TreeSSM state dimension	16
MLP expansion ratio	4
DropPath	0.1

**Table 4:** Performance comparison of different models.

Model	Accuracy (%)	Loss	Precision (%)	Recall (%)	F1-Score (%)	Params (M)	FLOPs (G)	Latency (ms)
S_ConvNeXt	99.15	1.810	99.12	99.06	99.09	28.59	4.47	4.85 ± 0.13
S_ViT	97.83	1.897	97.91	97.85	97.88	5.72	1.26	5.47 ± 0.84
S_PVT	99.19	1.830	99.18	99.14	99.16	13.23	1.94	5.78 ± 0.42
S_Bi_Mamba	99.08	1.816	99.03	99.00	99.01	7.15	1.08	16.75 ± 0.96
S_Mamba	99.53	1.767	99.51	99.47	99.49	37.13	2.92	11.59 ± 0.53
S_T_Mamba	99.78	1.658	99.68	99.66	99.67	29.96	4.78	60.63 ± 8.34

**Table 5:** Recognition accuracies for the different calf behaviors.

Model	Accuracy (%)				
	Diarrhea	Walking	Standing	Lying	Eating
S_ConvNeXt	100	96.88	99.09	99.55	99.78
S_ViT	100	93.70	96.22	99.60	99.73
S_PVT	100	97.31	98.90	99.55	99.95
S_Bi_Mamba	100	96.82	98.66	99.51	100
S_Mamba	100	99.23	99.62	99.73	99.78
S_T_Mamba	100	98.90	99.43	99.96	100

**Table 6:** Performance comparison of different models with and without sequence processing strategy.

Model	Accuracy (%)
ConvNeXt	63.47
S_ConvNeXt	99.15
ViT	77.45
S_ViT	97.83
PVT	78.29
S_PVT	99.19
Bi_Mamba	66.81
S_Bi_Mamba	99.08
T_Mamba	87.27
S_T_Mamba	99.78

**Table 7:** Performance comparison of different sequence frame lengths.

Model	5	10	15	20
S_ConvNeXt	84.45	91.47	96.97	99.15
S_ViT	87.99	93.20	95.88	97.83
S_PVT	92.66	96.79	98.45	99.19
S_Bi_Mamba	89.24	96.16	97.92	99.08
S_T_Mamba	96.83	99.31	99.75	99.78

**Table 8:** Performance comparison of different sampling strategies.

Model	Uniform	Random
S_ConvNeXt	98.69	99.15
S_ViT	97.62	97.83
S_PVT	98.91	99.19
S_Bi_Mamba	98.44	99.08
S_T_Mamba	99.74	99.78

## Data Availability

The dataset was developed by our research team and will be made publicly accessible upon reasonable request.
